# Doxorubicin Loaded Thermosensitive Magneto-Liposomes Obtained by a Gel Hydration Technique: Characterization and In Vitro Magneto-Chemotherapeutic Effect Assessment

**DOI:** 10.3390/pharmaceutics14112501

**Published:** 2022-11-18

**Authors:** Stefan Nitica, Ionel Fizesan, Roxana Dudric, Felicia Loghin, Constantin Mihai Lucaciu, Cristian Iacovita

**Affiliations:** 1 Department of Pharmaceutical Physics-Biophysics, Faculty of Pharmacy, “Iuliu Hatieganu” University of Medicine and Pharmacy, 6 Pasteur St., 400349 Cluj-Napoca, Romania; 2 Department of Toxicology, Faculty of Pharmacy, “Iuliu Hațieganu” University of Medicine and Pharmacy, 6A Pasteur St., 400349 Cluj-Napoca, Romania; 3 Faculty of Physics, “Babes-Bolyai” University, 1 Kogalniceanu St., 400084 Cluj-Napoca, Romania

**Keywords:** thermosensitive liposomes, doxorubicin, magnetoliposomes, magnetic hyperthermia, zinc ferrite nanoparticles, A459 cells

## Abstract

The combination of magnetic hyperthermia with chemotherapy is considered a promising strategy in cancer therapy due to the synergy between the high temperatures and the chemotherapeutic effects, which can be further developed for targeted and remote-controlled drug release. In this paper we report a simple, rapid, and reproducible method for the preparation of thermosensitive magnetoliposomes (TsMLs) loaded with doxorubicin (DOX), consisting of a lipidic gel formation from a previously obtained water-in-oil microemulsion with fine aqueous droplets containing magnetic nanoparticles (MNPs) dispersed in an organic solution of thermosensitive lipids (transition temperature of ~43 °C), followed by the gel hydration with an aqueous solution of DOX. The obtained thermosensitive magnetoliposomes (TsMLs) were around 300 nm in diameter and exhibited 40% DOX incorporation efficiency. The most suitable MNPs to incorporate into the liposomal aqueous lumen were Zn ferrites, with a very low coercive field at 300 K (7 kA/m) close to the superparamagnetic regime, exhibiting a maximum absorption rate (SAR) of 1130 W/gFe when dispersed in water and 635 W/gFe when confined inside TsMLs. No toxicity of Zn ferrite MNPs or of TsMLs was noticed against the A459 cancer cell line after 48 h incubation over the tested concentration range. The passive release of DOX from the TsMLs after 48h incubation induced a toxicity starting with a dosage level of 62.5 ug/cm^2^. Below this threshold, the subsequent exposure to an alternating magnetic field (20–30 kA/m, 355 kHz) for 30 min drastically reduced the viability of the A459 cells due to the release of incorporated DOX. Our results strongly suggest that TsMLs represent a viable strategy for anticancer therapies using the magnetic field-controlled release of DOX.

## 1. Introduction

One of the biggest challenges now facing chemotherapy, one of the most commonly used anti-tumor therapeutic approaches for cancer treatments, is represented by the controlled release of anti-cancer drugs targeted at a tumor area, as they are highly cytotoxic to healthy tissues [1,2]. Reducing systemic exposure to chemotherapeutic drugs is of paramount importance since it would drastically reduce the hard side effects on healthy tissues and the amount of anti-cancer drugs used during chemotherapy [3,4]. In the last decades, the possibility of using nanocarriers, encapsulating anti-cancer drugs, capable to circulate in the body to target the tumor and release their payload at controllable rates, has been extensively studied [5,6,7].

Nanocarriers based on magnetic nanoparticles (MNPs) are very promising for such use, as they can firstly be easily guided in the body and concentrated at the desired site by an extracorporeal magnetic field [8,9,10]. Secondly, the MNPs under the application of an alternating magnetic field (AMF) produce heat which can be exploited to sensitize the cancer cells (mild magnetic hyperthermia (MH)) and, at the same time, to trigger the fast release of the anti-cancer agents. Therefore, the synergistic effects of the simultaneous application of MH and chemotherapy, in the same nanocarrier-based MNPs, are expected to increase the effectiveness of cancer treatment.

A wide range of studies has focused on developing nanocarrier-based MNPs displaying a core-shell architecture, with the core being the MNPs. The shell was usually constituted from an organic polymer loaded with an anti-cancer drug. The organic polymer can be thermo-responsive, which means that it undergoes a conformational change at a specific temperature. Thus, the anti-cancer drug is retained at a physiological temperature within the shell and released as a consequence of MH that locally increase the temperature [11,12,13]. Often exploited also are pH-responsive polymers, which can release the anti-cancer drug due to the pH difference between blood/healthy tissues and tumors [14,15]. A much more robust shell is given by the mesoporous silica (SiO_2_) [16,17,18] or metal-organic frameworks (MOFs) [19,20,21], which are highly porous crystalline materials providing high porosity for encapsulating or incorporating anti-cancer drugs. 

One of the most attractive and studied nanocarrier systems for drug delivery is represented by liposomes (Ls) [22]. These nanocarriers present a spherical shape consisting of at least one lipid bilayer and an aqueous core, being capable of encapsulating either hydrophilic or hydrophobic anti-cancer drugs [23]. Nanoscale liposomal formulations, considered one of the most advanced delivery systems for cytotoxic drugs [24,25], have been used in clinical cancer therapy for many years, the most known example being the Doxorubicin (DOX)-loaded liposomes (Caelyx^®^, Schering-Plough; DOXIL^®^, OrthoBiotech) [26] with an improved pharmacokinetic profile and reduced cardiotoxic side effects as compared with the free drug [27]. Nevertheless, a much higher therapeutic efficacy of liposomal formulations is still needed and a large number of studies were conducted aiming at increasing the drug concentration at the target site by further enhancing their targeting and by localized triggering of the drug release from responsive liposomal formulations [28]. One of the main strategies used recently for reaching this goal is to use heat-activated liposomal drug carriers, lyso-thermosensitive liposomal doxorubicin (Thermodox^®^) being the first such system that has been evaluated in seven clinical studies [29].

The incorporation of MNPs in the Ls gives rise to the so-called magneto-liposomes (MLs), these structures being capable of controlled movement in the presence of a magnetic field gradient [30,31]. Regarding the aqueous MLs, the distribution of MNPs in the Ls’ structure defines three classes, in which MNPs are dispersed in the Ls’ aqueous core [32,33,34], embedded in the lipid bilayer [35,36] or attached to the Ls’ surface [37,38,39,40]. Recently, by combining MNPs and gold nanoparticles (NPs) inside the Ls’ aqueous core or distributed in different Ls compartments, plasmonic MLs have been created, with a great potential to achieve a synergistic effect between multiple treatment methodologies, such as chemotherapy, MH, and phototherapy [41,42,43,44]. 

For a controlled anti-cancer drug release, the MLs have been prepared by employing different formulations of thermo-sensitive lipids, thus resulting in thermo-responsive MLs (TsMLs) [45]. Similar to the case of thermo-responsive polymers, this class of MLs is characterized by a transition temperature (T_m_), above which the permeability of the lipid bilayer increases, thus facilitating the release of the anti-cancer drugs in a controlled manner [46,47]. The manufacturing processes of TsMLs are laborious, thus their clinical translation has been slowed down. Conventional preparation methods, such as thin film hydration [48,49,50,51,52,53,54], reverse-phase evaporation [55,56], and the double emulsion method [57] give rise to large and multilamellar TsMLs. To reduce their dimension (a few hundred nanometers in diameter) and obtain unilamellar TsMLs, different post-synthesis techniques must be applied, such as sonication, extrusion, and size-exclusion chromatography. The sonication process degrades or contaminates the sample, resulting in poor batch-to-batch reproducibility, while extrusion suffers from great product loss, obtaining a low production yield in the end. Therefore, there is a need to develop new synthesis protocols that allow efficient MNP loading into TsLs and eliminate the use of post-synthesis techniques [58,59].

In this paper, we have developed and optimized a simple and reproducible route for the synthesis of unilamellar TsMLs without the need for post-synthesis techniques. Moreover, the one-pot method allows the loading of the Ls with both the drug and the MNPs simultaneously. The MNPs, successfully entrapped in the liposomal aqueous core, consist of zinc ferrites that were fully investigated using multiple analytical methods such as transmission electron microscopy (TEM), X-ray diffraction (XRD), vibrating sample magnetometer (VSM), and magnetic hyperthermia (MH). The prepared TsMLs were characterized for physicochemical properties such as size, morphology, and DOX entrapment efficiency. Two independent cytotoxicity assays were applied to determine the cytotoxicity of TsMLs loaded with DOX on the human pulmonary carcinoma A549 cancer cell line. The in vitro DOX release and the magneto-chemotherapeutic effect under an alternating magnetic field (AMF) stimulus have also been examined. 

## 2. Materials and Methods

### 2.1. Materials

The synthesis of Zn ferrites has been done using the following chemicals: iron (III) acetylacetonate (≥98.00%, Merck Schuchardt OHG, Hohenbrunn, Germany), zinc (II) acetylacetonate (≥98.00%, Merck KGaA, Darmstadt, Germany), oleic acid (Sigma-Aldrich, Steinheim, Germany), tetra ethylene glycol (Carl-Roth, Karlsruhe, Germany, ≥99%) and benzyl ether (Sigma-Aldrich, Steinheim, Germany). The following lipids have been used in the preparation of TsMLs: 1,2-dipalmitoyl-sn-glycero-3-phosphocholine (DPPC) (Lipoid, Ludwigshafen, Germany), 1,2-distearoyl-sn-glycero-3-phosphocholine (DSPC) (Lipoid, Ludwigshafen, Germany), and cholesterol (Sigma-Aldrich, Steinheim, Germany). Additional chemicals used in the study were ethanol (Chemical, Iasi, Romania), hexane (Honeywell, Seelze, Germany), cyclohexane (Honeywell, Seelze, Germany), sodium periodate (Sigma-Aldrich, Steinheim, Germany), acetonitrile (Sigma-Aldrich, Steinheim, Germany), ethyl acetate (Chemical, Iasi, Romania), chloroform (Chemical, Iasi, Romania), ammonium sulfate (Sigma-Aldrich, Steinheim, Germany), doxorubicin hydrochloride (Sigma-Aldrich, Steinheim, Germany) and Osmium tetroxide (Merck Schuchardt OHG, Hohenbrunn, Germany) 

### 2.2. Synthesis of MNPs and Their Water Transfer

The Zn ferrites have been synthesized by means of the thermal decomposition method. Briefly, a mixture of 1.00 mmol of iron (III) acetylacetonate, 0.1 mmol of zinc (II) acetylacetonate, 2.9 mL of oleic acid, 1 mL of oleylamine, 1 mL of tetra ethylene glycol, and 20 mL benzyl ether were magnetically stirred at 50 °C with 500 rot/min for 1h (MR-HEi-TEC, Heidolph Instruments GmbH&Co.KG, Schwabach, Germany). Upon degassing with a flux of gaseous nitrogen for 5 min, the mixture was sealed in a stainless steel reaction vessel using a Teflon gasket and five screws. The reaction mixture was primely heated to 200 °C with at a rate of 6 °C/min using an oven (Nabertherm GmbH, Lilienthal, Germany) equipped with a temperature controller (JUMO dTron 316, JUMO GmbH & Co.KG, Darmstadt, Germany) and kept at that temperature for 2 h, after which the temperature was raised to 300 °C at a rate of 3 °C/min and kept at that temperature for an additional 1h. The resulting black product was separated with a neodymium magnet and washed several times using a 20 mL mixture of ethanol/hexane through ultrasonication (15 min) and magnetic separation. The purified Zn ferrites were stored in 20 mL cyclohexane for further processing.

Water transfer has been realized through the oxidation of oleic acid by sodium periodate. Briefly, 30 mL of aqueous sodium periodate solution (c = 60 mg/mL) was added with 10 mL of acetonitrile and ethyl acetate mixture (*v*:*v* = 1:1) and 10 mL of MNPs dispersed in cyclohexane. The mixture was mechanically stirred (Nahita, Auxilab S.L., Beriain, Spain) for 2 h until all MNPs passed from cyclohexane (top) into the water (bottom) phase. The MNPs were separated by a neodymium magnet and washed with double distilled water three more times. Finally, the hydrophilized MNPs were dispersed in the necessary volume of double distilled water to achieve a concentration of 4 mg_MNPs_/mL. The obtained aqueous dispersion was stored in a glass container.

### 2.3. Preparation of Thermosensitive Liposomes Loaded with MNPs and Doxorubicin

A mixture consisting of 30 mg of DPPC, 15 mg of DSPC and 5 mg of cholesterol (6:3:1 weight ratio) has been dissolved in a mixture containning 2 mL of chloroform and 3 mL of hexane, which has a density slightly lower than that of water. 1.5 mL of MNPs dispersion in 1M aqueous ammonium sulfate solution—was added to the lipidic solution. The mixture was then sonicated for 6 min using a Vibra-Cell™ Ultrasonic probe sonicator, model VCX 500 equipped with a tapered microtip of Ø 6 mm (Sonics&Materials, Inc., Newtown, CT, USA). The micro-emulsion formed was further introduced in the round bottom flask of a rotary-evaporator (Heidolph Instruments GmbH&Co.KG, Schwabach, Germany)). The installation parameters were set as follows: the flask rotation speed to 220 rot/min, the installation pressure to 600 mbar, and the water bath temperature to 45 °C. The mixture was kept in the installation for 10 min, resulting in the formation of a gel-like phase. The gel was further hydrated with 18.5 mL of double distilled water or an aqueous solution of doxorubicin hydrochloride—of desired concentration (either 10^−4^ M or 2 × 10^−5^ M). In the end, the mixture was introduced in the rotary-evaporator and vortexed at 45 °C for 5 min at 60 rot/min and a subsequent 10 min at 150 rot/min, resulting in TsMLs loaded with doxorubicin (DOX).

### 2.4. Characterization Methods

Transmission electron microscopy (TEM) images were obtained on a JEOL JEM-100CX II (JEOL, Tokyo, Japan) at 80 kV with a MegaView G3 camera (Emsis, Münster, Germany) running with Radius 2.1 software (Emsis). A drop of cyclohexane suspension of MNPs (10 μg_MNPs_/mL) was deposited on a carbon-coated copper grid, and we then waited for the solvent to evaporate. In the particular cases of TsLs and TsMLs, in 1 mL of liposomal aqueous solution (obtained by diluting 20 times the initial liposomal aqueous solution), a volume of 100 μL Osmium tetroxide was added to fix and stain the liposomes. After 30 min, a drop of the liposomal aqueous solution was deposited on a carbon-coated copper grid, and the excess water was removed by filter paper after 10 min. 

X-ray diffraction (XRD) patterns of powdered MNPs were collected with a Bruker D8 Advance diffractometer using Cu Kα radiation (Bruker AXS GmbH, Karlsruhe, Germany). The intensities were measured from 20° to 80° in continuous mode with a step size of 0.03° and a counting rate of 5 s per scanning step.

The UV–VIS absorption spectra of all samples were recorded with a T92+ UV–VIS Spectrophotometer PG INSTRUMENTS, using standard quartz cells at room temperature, over a spectral range between 400 nm and 600 nm and a spectral resolution of 2 nm. 

Hydrodynamic size and zeta potential measurements of samples (10 μg_MNPs_/mL) were determined using a Zetasizer Nano ZS90 (Malvern Instruments, Worcestershire, UK) in a 90° configuration, at room temperature. 

The magnetization curves of the MNPs were measured using a Cryogenic Limited (London, UK) vibrating sample magnetometer (VSM), operating at both 4K and 300K from 0 to ±4 T. 

The Specific Absorption Rate (SAR) was measured on a commercially available magnetic hyperthermia system, the Easy Heat 0224 from Ambrell (Scottsville, NY, USA) equipped with an 8-turn heating coil, made of a water-cooled copper tube, and a fiber-optic thermometer, placed in the middle of the sample volume, to measure the temperature value each second. The samples (0.5 mL of MNPs suspended in water, PEG 8K, or incorporated in TsLs at different concentrations) were placed in the center of the heating coil. The environment in close vicinity of samples was held at a physiological temperature of around 37 °C. The heating curves were recorded under a fixed frequency of 355 kHz at different alternating magnetic field (AMF) amplitudes: 10–60 kA/m. Details about the hyperthermia setup are provided elsewhere [60], and SAR calculations are provided in the Supplementary Materials (Section S1).

### 2.5. Cell Lines

The in vitro studies were conducted on a human pulmonary carcinoma A549 cell line, purchased from the American Type Culture Collection (ATCC, Manassas, VA, USA). Dulbecco’s Modified Eagle’s Medium (DMEM, Gibco, Paisley, UK) supplemented with 10% Fetal Bovine Serum (FBS, Gibco, Paisley, UK) was used to keep the cells at a temperature of 37 °C and 5% CO_2_ supplementation. The cell culture media was changed every other day, and the cells were used in the experiments once confluency of 80–90% was reached.

### 2.6. In Vitro Cytocompatibility Assays

Two different assays: Alamar Blue (AB) and Neutral Red (NR) assays were employed to assess the cytotoxicity of nanomaterials after 48 h of exposure to A549 cells seeded in 6 well plates. The cytotoxicity evaluation was performed on samples containing MNPs in a concentration of 15.625, 31.25, 62.5, 125, and 250 µg/cm^2^. After the exposure, cells were thoroughly washed with PBS and the AB and NR dyes were added. For the AB assay, cells were incubated for 3 h with a 200 µM resazurin solution, and the fluorescence was measured at an λ_excitation_ = 530/25 nm and λ_emission_ = 590/35 nm. For the NR assay, cells were exposed for 2 h to a 40 μg/mL filtered NR dye solution, and post-incubation, cells were washed to remove the non-internalized dye. The intra-cellular accumulated dye was further solubilized in a 50% hydroalcoholic solution containing 1% glacial acetic acid, and the fluorescence was measured at a λ_excitation_ = 530/25 nm and a λ_emission_ = 620/40 nm. For both assays, experiments were done in biological triplicates and the measurement of fluorescence was performed using a Synergy 2 Multi-Mode Microplate Reader.

### 2.7. Evaluation of Cellular Uptake

The cellular uptake of MNPs and TsMLs was quantitatively evaluated by the Liebig reaction of free Fe^3+^ with thiocyanate as described in the Supplementary Materials (Section S2) after incubation for 48 h. After the exposure, washed and trypsinized cells were centrifuged for 5 min at 4500× *g* and then processed for the Fe^3+^ quantification.

### 2.8. In Vitro Magnetic Hyperthermia

TsMLs and DOX-loaded TsMLs, suspended in a cell culture medium, were mixed with A549 cells that were previously seeded in 6-well plates for 24 h. Subsequently, the two aliquots of similar volume resulting from each mixture were centrifuged for 10 min at 100× *g* to reduce their volume to 0.5 mL. Upon removal of the excess supernatant, one of the aliquots was introduced in a water bath at 37 °C representing the negative control. The twin aliquot was exposed to an alternating magnetic field (AMF) for 30 min, working at a fixed frequency of 355 kHz and variable amplitudes of 10, 20, and 30 kA/m. Immediately after the AMF treatment, the cells from both aliquots were plated in 96-wells as six technical replicates. After 48 h, the cellular viability was measured by using AB and NR assays. Three biological replicates were considered for each experiment, while the data were normalized to the negative control.

### 2.9. Statistics

One-way Analysis of Variance (ANOVA) equipped with a post-hoc + Dunn’s test was used to analyze the data sets, while SigmaPlot 11.0 computer software (Systat) was employed to graphically represent the results, which are average values ± standard deviation (SD). Statistically relevant results were those showing *p* values < 0.05.

## 3. Results and Discussion

### 3.1. Characterization of Zinc Ferrites Nanoparticles and Their Heating Performances

**Figure 1:**
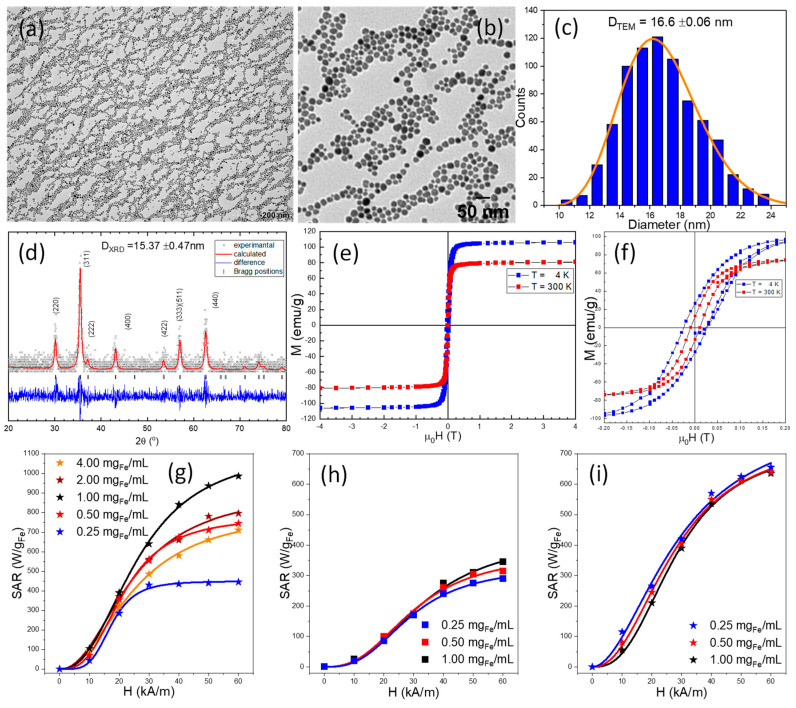
(**a**) Large scale and (**b**) zoom-in TEM image of Zn ferrites. (**c**) Size distribution histograms fitted to a log-normal distribution (orange line). (**d**) XRD diffraction patterns of Zn ferrites. (**e**) Hysteresis loops of Zn ferrites. (**f**) Low-field regime hysteresis loops of Zn ferrites. Specific absorption rate (SAR) dependence on the AMF amplitude (H) for Zn ferrites dispersed in (**g**) water and (**h**) PEG 8K at three different concentrations. (**i**) SAR dependence on H for TsMLs.

Small-sized magnetite MNPs (10 nm in diameter) have been generally used to fabricate TsMLs. This class of MNPs is mainly in a superparamagnetic state (SP) at room temperature and their heating performances under AMF are rather weak. Moreover, upon the confinement of MNPs inside the TsL, the heating properties are further reduced. Consequently, there is a need for MNPs with larger magnetic moments to enable the release of sufficient heat to destabilize the lipid bilayer membrane [61]. An already proven efficient way to increase the heating performances of magnetite MNPs is doping their structure with Zn cations [62,63,64,65,66,67]. We have used the well-known thermal decomposition technique to prepare Zn ferrites [65,68], in which the 1,2 hexadecanediol (a very expensive surfactant) has been replaced with tetra-ethylene-glycol (TEG). With 1 mL of TEG in the reaction mixture, the modified synthetic approach enables the formation of magnetite (Fe_3_O_4_) nanoparticles (NPs) of quasi-spherical shape and average diameter around 9 nm (Figure S3a,b). The addition of zinc (II) acetylacetonate in the reaction mixture produces well-defined spherical Zn ferrites with an average diameter of 16.6 nm, twice the diameter of Fe_3_O_4_ MNPs (Figure 1a–c). The X-ray diffraction pattern of Zn ferrites under 2θ values ranging from 20 to 80° are plotted in Figure 1d. The occurring diffraction peaks were ascribed to crystal planes: (220), (311), (400), (422), (511) and (440), respectively (JCPDS No. 88-0315) being similar to those of pure Fe_3_O_4_, indicating that Zn ferrites possess the crystal unit of a face-centered cubic spinel crystalline structure [62,63]. The Zn ferrites are single crystals as the average diameter from TEM images is close to the corresponding one given by XRD diffractogram, calculated using Scherrer’s formula by Gaussian fit of the peaks (220), (311), and (440). According to the hysteresis loop recorded at 4K (Figure 1e), the saturation magnetization (M_s_) is around 106 emu/g, higher than that of pure magnetite, confirming the location of Zn^2+^ ions in tetrahedral sites [69]. The spin canting effects, well-pronounced for MNPs of spherical shape, reduced the M_s_ at 300K with 26 emu/g. The low field hysteresis loops (Figure 1f) showed the presence of a coercive field (H_c_) of 25 mT (20 kA/m) at 4K, which is reduced to 9 mT (7 kA/m) at 300K, suggesting that the Zn ferrites are close to a superparamagnetic type behavior at the above temperature. 

The SAR dependence of Zn ferrites on the concentration and amplitude of the magnetic field is presented in Figure 1g for the concentration range 0.25–4.00 mg_Fe_/mL. The SAR dependence on the amplitude of the alternating magnetic field is sigmoidal and was fitted with a logistic function (Equations (S3) and (S4)), to obtain the SAR_max_ and the hyperthermia coercive field, i.e., the point of highest slope in the SAR = f(H_max_) curve [70]. The concentration dependence of SAR is nonmonotonic. SAR_max_ increases as the concentration increases in the range of 0.25–1.00 mg_Fe_/mL and decreases as the concentration further increases. The maximum SAR of 1130 W/g_Fe_ is reached for a concentration of 1 mg_Fe/_mL (Section S1 Table S1). As explained in our previous paper [66], the increase in the colloidal concentration drives the Zn ferrites from individual behavior to a collective one. In the concentration range of 0.25–1.00 mg_Fe_/mL, the interparticle interaction enhances their magnetization, while in the concentration range of 1.00–4.00 mg/mL, the dipolar interactions between the MNPs lead to a demagnetizing effect, reducing the magnetization of the colloid and thus the SAR_max_ [71]_._ As a consequence, the SAR_max_ values increase from 450 W/g_Fe_ at 0.25 mg_Fe_/mL to 1130 W/g_Fe_ at 1 mg_Fe_/mL and then decrease again to 810 W/g_Fe_ at 4 mg_Fe_/mL. Compared with Fe_3_O_4_ MNPs (Figure S3c), the Zn ferrites display a 3-fold higher SAR value due to the Zn doping. As expected, the random distribution of Zn ferrites in a solid matrix (PEG 8K) led to an important decrease of the SAR values: on average by 70% for 1 mg_Fe_/mL, 65% for 0.50 mg_Fe_/mL and 50% for 0.25 mg_Fe_/mL. For all three concentrations, the SAR values are almost identical in the H range of 10 to 30 kA/m. A small decrease of SAR values with decreasing the concentration can be identified starting with H of 40 kA/m. 

### 3.2. Preparation of Thermosensitive Magneto-Liposomes Loaded with Doxorubicin and Their Heating Performances

**Figure 2:**
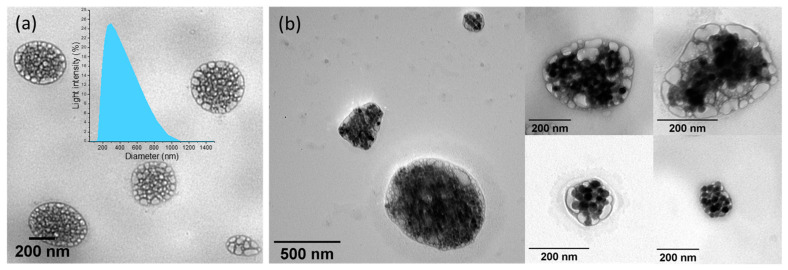
TEM images of (**a**) TsLs and (**b**) TsMLs. The inset represents the DLS spectrum of TsLs.

Normally, by selecting the phospholipids and adjusting their composition, thermosensitive liposomes (TsL) can be designed to display a leakage temperature range, within which the lipid bilayer membrane undergoes a phase transition from the crystalline form to a liquid one and cause the release of payload. In this work, we adopted the phospholipidic composition used by Anikeeva et al. [57]—with less cholesterol, that enables the formulation of TsL with a phase-transition temperature between 42–43 °C. Instead of the double emulsion method for liposome preparation, we used an osmotic-mediated hydration of the lipid gel method, as described in the Section 2. An ammonium sulfate aqueous phase containing Zn ferrites was transferred into a solution of thermosensitive lipids in a mixture of hydrophobic solvents. After the formation of the micro-emulsion through sonication, the hydrophobic phase was rapidly evaporated. The hydration of the formed gel with an aqueous solution of doxorubicin produced spherical TsMLs with an average diameter of 365 (±170) nm (Figure 2a). Various amounts of Zn ferrite were used in the preparation to achieve a maximum ratio between encapsulated and empty TsLs. We found that an amount of 10 mg_MNPs_ in the microemulsion mixture is adequate to obtain a high number of TsLs containing Zn ferrites, with a small fraction (less than 5%) of empty TsLs. All Zn ferrites were encapsulated inside the TsLs as no free MNPs were observed on the TEM images. However, above this threshold, some Zn ferrites remain outside of TsLs within the supernatant of the liposomal solution. Room temperature TEM analysis clearly shows that the Zn ferrites are homogeneously distributed inside the inner aqueous core of the TsLs (Figure 2b). The Zeta potential of Zn ferrites upon water transfer was found to be −37.28 mV, this negative value being due to the carboxyl groups resulting upon oxidation of oleic acid by sodium periodate [65,72]. The incorporation of Zn ferrites inside the aqueous core of TsLs led to TsMLs exhibiting a less negative Zeta potential of −13.47 mV. 

The preparation method of TsMLs developed herein is very efficient as basically all MNPs, below a certain threshold, are embedded in the liposomal pool, eliminating the need for post-synthesis purification methods. Moreover, the size of TsMLs of a few hundred nanometers directly resulting from synthesis, without the use of downsizing techniques, is adequate for biomedical applications. It is worth mentioning that the concentration of ammonium sulfate from the solution used for emulsion preparation played an important role in the gel hydration efficiency, the optimal value being situated at 1M in our experiments.

To check the suitability of our preparation method, we have extended it to other types of MNPs. The small Fe_3_O_4_ NPs, presented above are incorporated in the liposomal pool resulting in well-defined TsMLs (Figure S4a). Instead, 27 nm ferromagnetic Zn ferrites with higher SAR (>3000 W/g_Fe_) [65] tend to form chains and avoid entrapping in the liposomal pool (Figure S4b). Therefore, it appears that the successful preparation of TsMLs is restricted to using SP-MNPs or MNPs close to the superparamagnetic regime. In the third case, we have coated the current Zn ferrites in a silica shell, according to a procedure published previously [65] in small clusters containing few MNPs. According to TEM analysis, there is no embedment in the liposomal pool: the silica-coated Zn ferrites are located either outside the TsLs or are attached to the lipid bilayer (Figure S4c). Both Zn ferrites and Fe_3_O_4_ NPs exhibit a higher negative Zeta potential as compared to silica-coated ones. It results that negatively charged MNPs are prone to be entrapped in the liposomal pool, probably due to a possible interaction with the positive charges of the zwitterionic lipids (the ammonium group from phosphatidylcholine) occurring during the preparation. 

The amount of DOX encapsulated in TsMLs was estimated by UV-Vis spectroscopy based on a calibration curve that exhibits the DOX concentration as a function of the absorbance at 495 nm (Figure S5). Upon preparation of the TsMLs with DOX, the liposomal solution was magnetically decanted, and the supernatant was used to determine the non-encapsulated DOX amount. The difference between the initial DOX amount and that of non-encapsulated DOX gives the amount of encapsulated DOX. Two different starting concentrations of DOX have been used: 10^−4^ M and 2 × 10^−5^ M corresponding to 930.47 and 178.29 µg of DOX respectively. According to Table 1, for the high initial DOX concentration, the amount of encapsulated DOX was 392 µg, resulting in an encapsulation efficiency (EE = DOX_encapsulated_/DOX_inital_ × 100) of 42%. The EE decreases to 39% for low initial DOX concentration, the encapsulated DOX amount being 70 µg, representing an amount 5.6 times lower than the first case.

The release of encapsulated doxorubicin from TsMLs crucially depends on the capabilities of Zn ferrites, confined in TsLs, to produce heat under AMF stimulus. Therefore, in the next step of our study, we have examined the heating capabilities of well-designed TsMLs. As presented in Figure 1i, for a concentration of 1.00 mg_Fe_/mL, the SAR values exhibit a sigmoidal increase from 55 W/g_Fe_ to 635 W/g_Fe_ with increasing H from 10 to 60 kA/m. For the entire H range, the decrease in concentration led to only a very slight increase in SAR values (Figure 1i), contrary to previous cases for unconfined or immobilized Zn ferrites (Figure 1g,h) for which the concentration dependence of SAR is much more pronounced. This situation is similar to the case of Zn ferrites coated in an SiO_2_ layer in relatively small clusters [65], which also present a very slight dependence of SAR on concentration. In both cases, with the MNPs confined in the liposomes or clustered in a silica shell, the SAR values are smaller as compared to the water dispersion case. This is explained by the strong dipolar interaction between the MNPs within the TLs or the cluster. However, the fact that increasing the number of clusters and thus reducing the main distance between them has very small or no influence on SAR is a clear indication that inter-cluster or inter-liposomal interactions are negligible [66] and either the lipidic bilayer or the silica shell avoids the confined Zn ferrites to come into contact and form bigger aggregates as the concentration increases. Thus, the inter-TsMLs interaction energy is negligible and the increase in the number of TsMLs in a given volume will not affect the SAR. Compared with the case where the Zn ferrites are dispersed in water, their confinement in the aqueous lumen of TsLs led to a reduction of SAR values. As mentioned above, this decrease can be explained by an increase in the dipolar interaction between the MNPs confined inside the TsMLs. As can be seen from Table S1, the SAR values for the water dispersions decrease from 1130 W/g_Fe_ at 1mg_Fe_/mL to 810 W/g_Fe_ at 4 mg_Fe_/mL. Based on the initial concentrations of MNPs and the final volumes obtained, the actual concentration of MNPs inside a TsLs is 6–7 mg_Fe_/mL or higher, and this concentration increase is a reasonable explanation for SAR_max_ values of 700 W/g_Fe_ for the TsMLs.

With respect to immobilized Zn ferrites, the SAR values of Zn ferrites confined in TsMLs are higher for all H (Tables S1 and S2). This can be due to a certain mobility of TsMLs generating heat through physical rotation. We checked if this partial mobility allows the MNPs to align themselves in a static DC field aiming at increasing the SAR. In our recent paper, we have shown that a direct current bias magnetic field (H_DC_) applied parallel or perpendicular to AFM lines enhances the SAR mainly at higher H for water-dispersed MNPs [66]. Therefore, an H_DC_ of 10 kA/m has been applied parallel to AMF lines during MH experiments in the second set of experiments performed on TsMLs. According to Figure S6, for all three concentrations and all H_max_ values, the SAR values are smaller than those recorded without H_DC_, while the H_cHyp_ values are shifted toward higher H (Table S2). This type of behavior is similar to that recorded for immobilized Zn ferrites [66]. Thus, by confinement inside the aqueous lumen of TsLs, the Zn ferrites are partially immobilized, being restricted to organize in chains under the influence of either field. 

### 3.3. Cellular Viability of Doxorubicin-Loaded TsMLs

**Figure 3:**
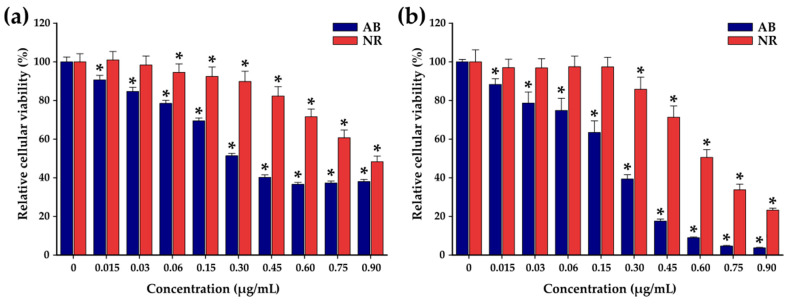
Cytocompatibility of doxorubicin on A549 cell line after (**a**) 24 h and (**b**) 48 h exposure. Data are presented as relative values to the negative control (100%), as mean ± SD of three biological replicates. The significant differences compared to the negative control (ANOVA + Dunn’s; *p* < 0.05) are noted with asterisks (*).

Before evaluating the synergistic effect of DOX-loaded TsMLs upon MH treatment, in our study, we have determined the anti-tumoral effect (IC_50_) of doxorubicin (DOX) on the A549 cell line. Cellular viability of A549 cells was measured by Alamar Blue (AB) and Neutral Red (NR) assays after exposure to various concentrations of DOX (ranging from 0.0145 to 0.870 µg/mL) for 24 and 48 h. The AB assay shows that the DOX presents a cytotoxic effect, after 24 h, starting with a concentration of 0.03 µg/mL. The cell viability decreases with increasing the DOX concentration, reaching a plateau of around 36% for the last three tested concentrations (Figure 3a) with an IC_50_ of 0.3 μg/mL, similar to other reports in the literature for A459 cells [70]. By doubling the incubation time to 48 h, the cell viability continued to decrease for all concentrations (Figure 3b). The drop in cell viability was more pronounced at higher tested concentrations. The IC_50_ was shifted from 0.3 µg/mL at 24 h to 0.18 µg/mL at 48 h. The NR assay, which is less sensitive than the AB assay, also indicated a pronounced cytotoxic effect of DOX on A549 cells at 48h. Since DOX presents a relevant anti-tumoral effect towards A549 cells at 48 h, the safe working concentrations of the three Zn ferrites, TsMLs and DOX-loaded TsMLs were established at this incubation interval time.

**Figure 4:**
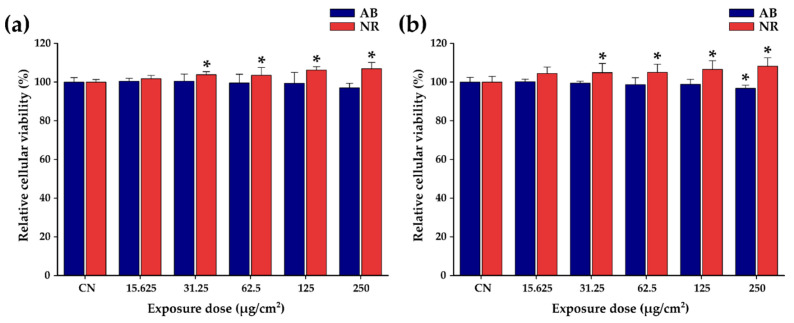
Cytocompatibility of (**a**) Zn ferrites and (**b**) TsMLs on A549 cell line after 48h exposure. Data are presented as relative values to the negative control (100%), as mean ± SD of three biological replicates. The significant differences compared to the negative control (ANOVA + Dunn’s; *p* < 0.05) are noted with asterisks (*).

As Figure 4a,b show, the lack of cytotoxic effects after 48 h of exposure of A549 cell lines to the Zn ferrites and TsMLs over the entire concentrations range (15.625 to 250 µg/cm^2^) can be noted. The lack of cytotoxic effect can be explained by the poor internalization of both Zn ferrites and TsMLs (Figure S7). After 48 h, the relative internalization of Zn ferrites was low: ranging between 2.7% and 25% for Zn ferrites alone and between 2.3–18% for Zn ferrites entrapped in TsLs (Figure S7). For both cases, the relative internalization decreased by increasing the exposure dose (Figure S7). These small percentages of relative internalization mean a very low amount of internalized MNPs. In each case, the highest internalized amount was 20 µg_Fe_ per well (around 20 pg_Fe_ per cell), which is well below the toxicity threshold of MNPs. The lack of internalization can be explained by the negative surface charge of Zn ferrites and TsMLs which are hardly endocytosed by A549 cells, as their membrane displays extensive negatively charged domains.

**Figure 5:**
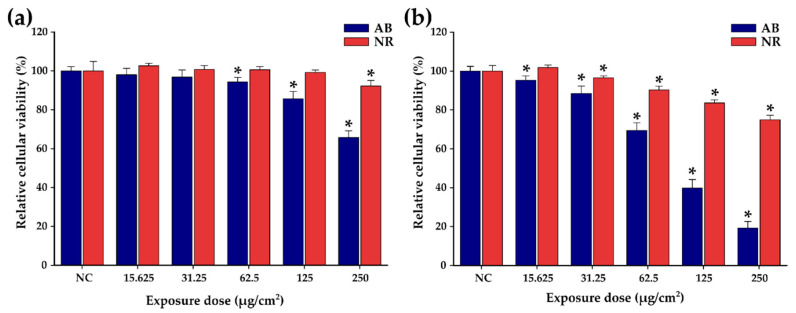
Cytocompatibility of (**a**) l-DOX-TsMLs and (**b**) h-DOX-TsMLs on A549 cell line after 48h exposure. Data are presented as relative values to the negative control (100%), as mean ± SD of three biological replicates. The significant differences compared to the negative control (ANOVA + Dunn’s; *p* < 0.05) are noted with asterisks (*).

In contrast to previous observations obtained for Zn ferrites either alone or entrapped in TsLs, the exposure of A549 cells to the TsMLs loaded with DOX caused a decrease in the cell viability of A549 cells (Figure 5). After 48h of exposure to l-DOX-TsMLs (TsMLs loaded with a small amount of DOX), the NR assay showed no toxicity over the entire concentration range (Figure 5a). Based on the fact that the concentration of the tested nanomaterial can be described as non-toxic if the cells’ viability is above 80%, the AB assay indicated a concentration threshold of 250 µg/cm^2^, where a drop in the cell viability to 65% was recorded (Figure 5b). In the case of h-DOX-TsMLs (TsMLs loaded with a high amount of DOX), the NR assay indicated toxicity at a concentration of 250 µg/cm^2^ (Figure 5b). Instead, the AB assay showed a more pronounced toxicity effect, starting with a concentration of 62.5 µg/cm^2^, where a cell viability of 69% was reached (Figure 5b). By increasing the concentration of h-DOX-TsMLs, the cell viability decreased considerably, reaching 19% at the highest tested concentration (250 µg/cm^2^). Since the TsMLs without DOX are not toxic over the tested concentration range, the decrease in the cell viability may suggest that the DOX was released from the TsMLs during the incubation with A549 cells through a passive flux. Samples containing the same amount of h-DOX-TsMLs have been incubated without cells for 48 h to quantify the amount of DOX passively released from TsMLs. By applying the second protocol for DOX concentration determination (Section S5), we were able to detect a DOX concentration of 1.8–2.1 µg/mL in the samples containing the highest amount of h-DOX-TsMLs (dosage of 250 µg/cm^2^), which represent roughly 10% of the encapsulated DOX concentration. For the other samples, the amount of DOX passively released was under the detection limit of the employed spectroscopic method, which is around 1.5 µg/mL. 

### 3.4. In Vitro Magnetic Hyperthermia

**Figure 6:**
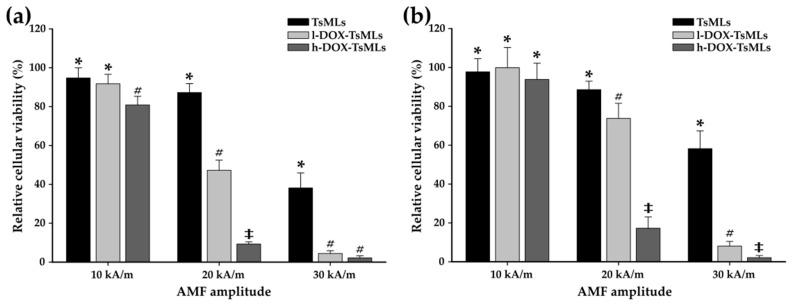
Cytotoxic effects of TsMLs with and without DOX, mixed with A549 cells were evaluated after a 30 min exposure to AMF of 355 kHz and amplitudes of 10, 20, and 30 kA/m. Cellular viability was measured using Alamar Blue (**a**) and Neutral Red (**b**) assays and presented as the mean ± SD of three biological replicates. Data are presented as relative values to their negative control (100%). Different symbols(*,#,‡) indicate a statistically significant difference between the groups (ANOVA + Holm-Sidak; *p* < 0.05).

**Figure 7:**
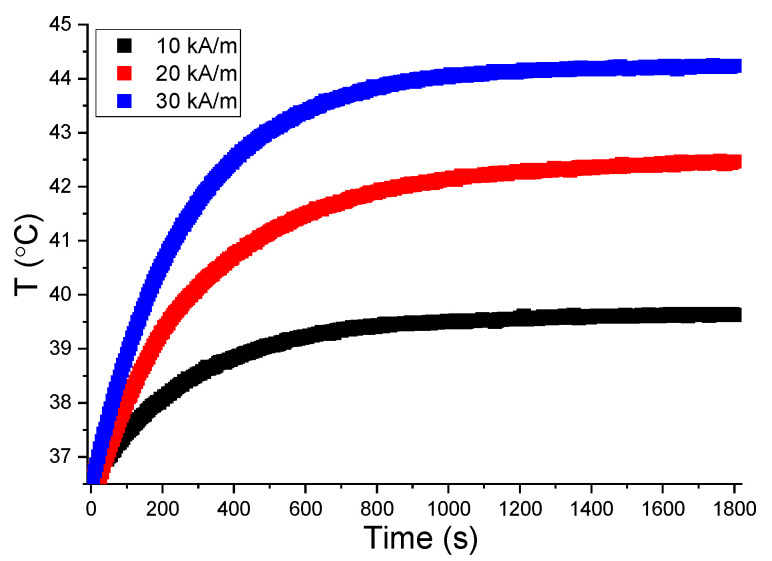
Typical heating curves of TsMLs mixed with A549 cells at a concentration of 0.2 mg_Fe_/mL in a volume of 500 μL at different H_max_ values: 10, 20, and 30 kA/m at a constant frequency of 355 kHz.

According to the cytotoxicity studies, the threshold concentration of h-DOX-TsMLs below which there is no induced toxicity by the passively released DOX is 31.25 µg/cm^2^. This dosage corresponds to 0.2 mg_Fe_/mL. In the first step of in vitro MH experiments, the A549 cells were mixed with TsMLs in a concentration of 0.2 mg_Fe_/mL and exposed for 30 min to AMF of 10, 20, and 30 kA/m, to check whether or not the heat released by the encapsulated MNPs will affect the cell integrity. As can be seen in Figure 6a,b, both assays indicated no toxicity when exposing the mixture (A549cells+TsMLs) to an AFM of 10 and 20 kA/m. This is in accordance with the small values of saturation temperature reached upon AFM exposure: 39.6 ± 0.3 °C for 10 kA/m and 42.4 ± 0.2 °C for 20 kA/m (Figure 7). A statistical decrease in the A549 cell viability is observed with both assays (38% for AB and 58% for NR) upon exposure to 30 kA/m (Figure 6a,b). The saturation temperature reached during AMF exposure was 44.2 ± 0.2 °C (Figure 7), which is above the temperature at which the A549 cells, exposed for 30 min to MH treatment, received a 50% lethal dose (LD50%) [65]. 

The same experiments were repeated with l- and h-DOX-TsMLs in the mixture. At 10 kA/m the l-DOX-TsMLs did not induce any toxicity to A549 cells (Figure 6a,b). A comparison between the groups based on the AB data indicated a statistical difference between h-DOX-TsMLs and the other two treatments (l-DOX-TsMLs and TsMLs) (Figure 6a), while no difference was observed based on the NR data (Figure 6b). By increasing the AMF amplitude to 20 kA/m, lower cellular viability was measured in all three conditions, with a more prominent decrease being observed in the case of DOX-loaded TsMLs (Figure 6a,b). Similar to the previous observation, the AB assay indicated stronger toxicity than the NR assay. At this amplitude, the recorded viabilities were 87, 47, and 9% for the TsMLs, l- and h-DOX-TsMLs, respectively, with significant differences being observed between all groups (pairwise comparisons). As the recorded temperatures were almost identical, these values indicate that the release of DOX from the TsMLs is responsible for the higher cytotoxicity observed in the case of DOX-loaded TsMLs, the toxicity being also dependent on the DOX loading of the TsMLs. A statistically significant difference between the groups was also observed using the NR assay; however, the recorded viabilities were slightly higher (Figure 6b). By further increasing the amplitude of the AMF to 30 kA/m, the recorded viabilities decreased in all three cases. Similar to the results observed at 20 kA/m, significant differences were observed between the DOX-loaded and unloaded TsMLs, the recorded viabilities being less than 10% from the control values in both assays for the DOX-loaded TsMLs (Figure 6a,b). The Two-Way ANOVA test having as variables, the type of TsMLs and the AMF amplitude, indicated that both factors influence the measured viability resulted from both AB and NR assays. 

Overall, the performed experiments indicate that the decreased cellular viability can only be caused by the DOX flux released from TsMLs structure under the oscillating magnetic field. Moreover, as the negative control of each sample was represented by the same number of cells exposed to the loaded or unloaded TsMLs, but not to the AMF, the current results are not the result of a passive flux of DOX from the TsMLs during the 48 h incubation period.

For a more straightforward explanation of the biological effects observed, we attempted to quantify the release of DOX from the l- and h-DOX-TsMLs under the influence of the AMF. In the case of h-DOX-TsMLs, the amount of encapsulated DOX was 8.1 µg/mL per sample, allowing the quantification of the released DOX from TsMLs after the MH treatment. Upon 30 min MH exposure of h-DOX-TsMLs to 30 kA/m, 55% of the DOX load was released (4.4 ± 0.3 µg/mL). For 20 kA/m, the released DOX amount was 2.8 ± 0.3 µg/mL, representing 35% of the DOX load, while for 10 kA/m no DOX released was measured. These results support the cytotoxicity observed after AMF treatment, as at these concentrations the cytotoxic effects of doxorubicin are present (Figure 3). Since the amount of encapsulated DOX in the l-DOX-TsMLs at a concentration of 0.2 mg_Fe_/mL was 1.4 µg/mL, close to the detection limit of the employed spectroscopic method, the amount of released DOX could not be quantified. However, taking into consideration the releases observed from h-DOX-TsMLs, a high enough quantity of DOX (0.8 µg/mL and 0.5 µg/mL at a 55% and 35% release) would be attained to induce cytotoxic effects on the cancerous cells, thus explaining the current results, and the difference between the more potent cytotoxic effects of l-DOX-TsMLs in comparison with TsMLs upon MH treatment. 

Since both high-frequency and high-amplitude AMF produce eddy currents in conducting media that can lead to damage to the human body, human exposure to AMF sets a safety limit on the frequency and amplitude of the AC magnetic field, the product between these two parameters should be 5 × 10^9^ Am^−1^s^−1^ [73]. It was demonstrated recently that this safety limit can be extended to a product of 9.59 × 10^9^ Am^−1^s^−1^ [74]. Our AFM parameters of 20–30 kA/m and 355 kHz, which led to an A549 cell survival reduction by more than 90% upon 30 min exposure, fall in the new safety limit, indicating that the MH applicability of our DOX-TsMLs might be extended to in vivo experiments. DOX release from TsMLs reaching a cell death rate as high as 83% was also recently demonstrated by Forte Brolo et al. [40]. Although their AMF parameters defined an Hf product of 4.85 × 10^9^ Am^−1^s^−1^, which is smaller than ours (Hf = 7–10.65 × 10^9^ Am^−1^s^−1^), the AMF was applied for 1h. A similar time interval has been applied by Pradhan et al. [75] in their study on HeLa cells targeted by folate-DOX-TsMLs, resulting in less than 7% cell viability with an Hf product of 3.5 × 10^9^ Am^−1^s^−1^; however, the DOX concentration was twice that in our case. Complete elimination of HeLa cells by combined MH and chemotherapy has also been realized by Shah et al. [76], with an Hf product of 5 × 10^9^ Am^−1^s^−1^ and an exposure time of 30 min; the amount of DOX-TsMLs in the samples being 10 times higher than in our case. 

A key parameter for the successful application of DOX-TsMLs in both in vitro and in vivo experiments is the heating capabilities of MNPs. For a Hf product satisfying the safety limit, the MNPs should deliver sufficient heat in a short time to reach the T_m_ of TsMLs for releasing the DOX. Our Zn ferrites have excellent heating performances, individually dispersed in water or even confined in the liposomal pool, which enable us to reach T_m_ at a very low concentration (0.2 mg_Fe_/mL) in 15 min (Figure 7). It is worth mentioning that we were able to induce an A549 cell death rate of 45% and 85% by exposing samples containing h-DOX-TsMLs and A549 cells for 15 min to 20 and 30 kA/m, respectively. In addition, compared to previous studies, the preparation protocol developed herein is very simple and quite fast, and the amount of resulting DOX-TsMLs is larger due to the high Zn ferrites’ entrapping efficiency. Since the TsMLs have a short lifetime (three-four days), all of these issues are of paramount importance for subsequent in vivo studies and clinical trials, which require large quantities of DOX-TsMLs. 

## 4. Conclusions

In summary, the presented results prove the successful obtaining of thermosensitive magneto-liposomes capable of drug encapsulation and release under the influence of an alternating magnetic field. The employed synthetic procedure was fast, cost-effective, and reproducible, and consisted of two main steps: (1) lipid gel formation; and (2) osmotic-driven lipid gel hydration plus ammonium gradient-driven drug incorporation. The obtained thermo-sensitive magneto-liposomes were nano-sized, of spherical shape, homogeneous, and presented a high MNP_S_ entrapping efficiency. Thus, no further post-treatment was needed for improving the liposomal system’s physical characteristics. The effect of the formulation parameters such as the solvents used for dissolving lipids, the ratio between lipds, Zn ferrites and (NH_4_)_2_SO_4_ concentrations of the aqueous dispersion used for water-in-oil emulsion preparation, solvent temperature, pressure, and vortex speed employed in the gel formation step, were investigated and optimized. 

The SAR values of Zn ferrites exhibited a sigmoidal increase with H and strongly depend on the colloidal concentration, the maximum SAR of 1130 W/g_Fe_ being achieved for a concentration of 1 mg_Fe_/mL. The MH experiments of TsMLs showed that the SAR values of Zn ferrites decreased for each H, exhibiting a similar sigmoidal dependence on H, and did not vary within the colloidal concentration.These experimental observations represent further proof of the Zn ferrites confinement as small clusters within the liposomal core.

Due to the poor internalization in A549 cells, both Zn ferrites and TsMLs are not toxic upon 48 h incubation time over the studied concentration range (15.625 to 250 µg/cm^2^). In the absence of an AMF, the TsMLs loaded with doxorubicin exhibited a toxic effect due to the passive release of small drug amounts during the 48 h incubation time. When exposed to AMF, the doxorubicin-loaded TsMLs exhibited enhanced toxicity due to the release of higher drug amounts compared to the previous ones. 

The synthesis procedure described in this paper may be extended to incorporate multiple classes of MNPs and drugs, either hydrophilic or hydrophobic, and may present a practical interest due to its simplicity, effectiveness and high yield.

## Figures and Tables

**Table 1:** Loading capacity of TsMLs with doxorubicin.

Liposomes	Initial DOX Concentration	Initial DOX Amount	Non-Encapsulated DOX Amount	Encapsulated DOX Amount	Encapsulation Efficiency
h-DOX-TsMLs	10^−4^ M	930 ± 26 µg	538 ± 36 µg	392 ± 11 µg	42%
l-DOX-TsMLs	2 × 10^−5^ M	178 ± 19 µg	109 ± 28 µg	70 ± 9 µg	39%

## Data Availability

Not applicable.

## Supplementary Materials

The following supporting information can be downloaded at: https://www.mdpi.com/article/10.3390/pharmaceutics14112501/s1, Section S1: Details regarding hyperthermia setup, SAR calculation, and fitting the SAR = f(H_max_) curves; Figure S1: Heating curves for Zn ferrite MNPs dispersed in water and their fit with the Box-Lucas eq.; Table S1: Fitting parameters for SAR evolution with H_max_ for samples dispersed in water and immobilized in PEG 8K; Section S2: Iron concentration determination; Figure S2: Calibration curve for the iron concentration determination; Figure S3: TEM image, size distribution and SAR dependence on AMF amplitude of iron oxide MNPs; Figure S4: TEM images of TsMLs loaded with small superparamagnetic iron oxide MNPs, high coercivity Zn ferrite MNPs and silica-coated Zn ferrite MNPs; Section S5: Calculations for the determination of the doxorubicin concentrations in liposomes; Figure S5: UV-Vis absorption spectrum of doxorubicin and calibration curve for the concentration determination; Figure S6: SAR dependence on the AMF amplitude for the TsMLs with and without a superposed 10 kA/m static magnetic field; Table S2: Fitting parameters of the SAR evolution with H for TsMLs with and without the static DC magnetic field; Figure S7: Cellular internalization of the Zn ferrite MNPS in A459 cells after 48 h exposure.
