# Supplementary material for: Doxorubicin Loaded Thermosensitive Magneto-Liposomes Obtained by a Gel Hydration Technique: Characterization and In Vitro Magneto-Chemotherapeutic Effect Assessment
Source: Pharmaceutics. 2022 Nov 18;14(11):2501. doi: 10.3390/pharmaceutics14112501 (PMC9697793; doi:10.3390/pharmaceutics14112501)
Supplement: Supplementary file 1 [file pharmaceutics-14-02501-s001.zip › pharmaceutics-2022124-supplementary.pdf]

Supplementary Materials

Doxorubicin loaded thermosensitive magneto-liposomes obtained by a gel hydration technique: characterization and in vitro magneto-chemoterapeutic assessment

Stefan Nitica[1 ‡], Ionel Fizesan[2‡], Roxana Dudric[3], Felicia Loghin[2], Constantin Mihai Lucaciu[1*] and Cristian Iacovita[1]

[1] *Department of Pharmaceutical Physics-Biophysics, Faculty of Pharmacy, "Iuliu Hatieganu" University of Medicine and Pharmacy, 6 Pasteur St., 400349 Cluj-Napoca, Romania*

[2] *Department of Toxicology, Faculty of Pharmacy, "Iuliu Hațieganu" University of Medicine and Pharmacy, 6A Pasteur St., 400349 Cluj-Napoca, Romania*

[3] *Faculty of Physics, "Babes Bolyai" University, 1 Kogalniceanu St., 400084 Cluj-Napoca, Romania*

* Correspondence: clucaciu@umfcluj.ro; Tel: 0040-744-647-854 (M.C.L.)

‡These authors contributed equally.

S1. Magnetic hyperthermia:

The specific absorption rate (SAR) is defined as the heat released from a suspension of MNPs in unit time reported to the mass of iron content. It was used to quantify the heat performance of MNPs. For reliable determination of SAR, the temperature change ΔT versus time curves - where ΔT = T(t) − T0; T(t) is the temperature at time t and T_0 = 37 °C -, have been fitted with the Box-Lucas equation (Figure S1):

$$\Delta T = \frac{S_m}{k}\left(1 - e^{-k(t-t_0)}\right) \qquad \text{(S1)}$$

where the fitting parameters S_m and k are the initial slope of the heating curve and the constant describing the cooling rate, respectively. Thus, SAR can be calculated as:

$$\text{SAR} = \frac{c\, m\, S_m}{m_{Fe}} \qquad \text{(S2)}$$

where c is the specific heat of the colloid (in our case was approximated with the specific heat of water: $c = 4186\, J/kgK$ and PEG8K: $c = 2136\, J/kgK$ the MNPs contribution to the specific heat being negligible), $m = \rho/V$ is the mass of colloid, taken as the product between the density ($\rho_{water} = 0,997\ g/cm^3$ for water and $\rho_{PEG8K} = 1,125\ g/cm^3$ at 298K) and the volume. The iron concentration of samples was determined using the thiocyanate assay described in the section below. For the case of MNPs dispersed in water, prior to each measurements the sample has been sonicated for 10 seconds to assure a good colloidal dispersion over the entire aqueous volume. Each SAR value is a mean of three measurements realized on three different samples. For immobilized MNPs in PEG 8K, three different samples have been measured for each concentration. For Zn ferrites confined in thermosensitive liposomes, for each H, a distinct sample containing 0.5 mL magneto-liposomal aqueous suspension at desire concentration has been used. The SAR determination was done in triplicates.

The sigmoidal evolution of our experimental SAR data with H was well fitted (R^2 > 0.999) phenomenologically with a simple logistic function:

$$SAR = SAR_{max} \frac{\left(\frac{H}{H_{cHyp}}\right)^n * \propto}{1 + \left(\frac{H}{H_{cHyp}}\right)^n * \propto} \qquad (S3)$$

with:

$$\propto = \frac{n+1}{n-1} \qquad (S4)$$

where SAR_{max} - the saturation value of the SAR, H_{cHyp} - the hyperthermia coercive field, the value of the H for which the function presents the highest slope or the H at which the first derivative of SAR against H presents a maximum, and the exponent n – which indicate how

steep is the dependence of SAR on H. The values of these parameters for all four types of MNPs at each iron concentration are provided in Table S1 below.

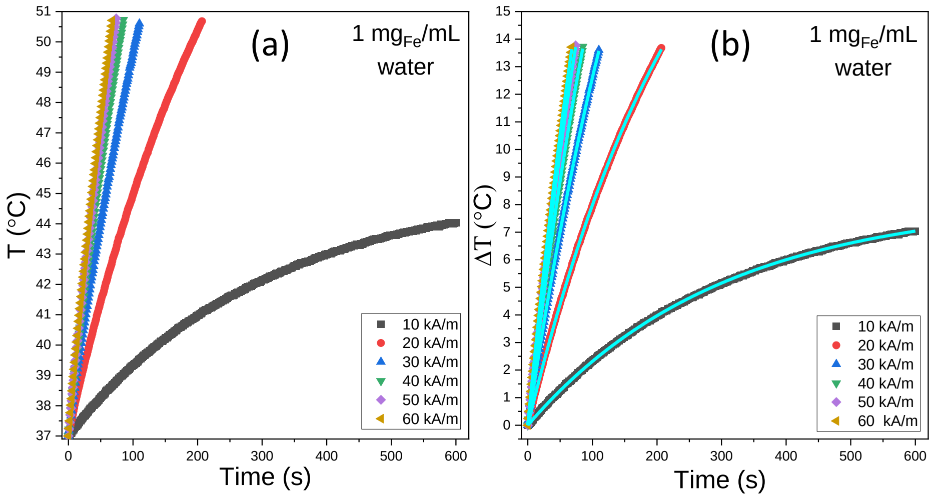

Figure S1. (a) The heating curves and (b) their corresponding temperature change ΔT versus time curves fitted with Box-Lucas equation (blue curves) of Zn ferrites, dispersed in water at an iron concentration of 1.00 mg_{Fe}/mL, recorded as a function of H (10 – 60 kA/m, step of 10 kA/m) at frequency of 355 kHz.

Table S1. Fitting parameters of SAR evolution with H for Zn ferrites dispersed in water and immobilized in PEG 8K.

**Dispersion medium**	**c (mg_{Fe}/mL)**	**SAR_{max} (W/g_{Fe})**	**H_{cHyp} (kA/m)**	**Power Coefficient n**
water	4.00	810	15.41	2.1
	2.00	910	15.53	2.2
	1.00	1130	18.16	2.4
	0.50	780	16.8	2.9
	0.25	450	15.6	4.4
PEG 8K	1.00	430	23.99	2.5
	0.50	375	22.29	2.6
	0.25	330	22.33	2.8

S2. Iron concentration determination:

The iron content of samples was measured using the thiocyanate assay. The same amount of magnetite (Fe_3O_4) and Zn ferrites powders (5 mg) have been dissolved in 5 mL of double distilled water through sonication. 1 mL of each colloidal suspension were magnetically separated and further suspended in 10 mL of HCl 12% solution for digestion at 80°C for at least 4 h. The incubation was followed by a centrifugation at 12000 g for 10 mins and the supernatants were collected for Fe^{3+} quantification. All iron species were oxidized to Fe^{3+} by incubating 50 µL of the supernatant with 50 µL of 1% ammonium persulfate for 30 mins. the colored Fe^{3+}-thiocyanate compound was obtained by adding 100 µL of 0.1 M potassium thiocyanate and the absorbance was measured at a λ = 490 nm using the Synergy 2 Multi-Mode Microplate Reader. The Fe^{3+} content of NPs was calculated from a Fe^{3+} standard curve with concentrations ranging between 2.5 - 140 µg/mL (Figure S2). For the magnetite nanoparticles the iron percentage was 71.8% closed to the theoretical value of 72.4%. In the case of Zn ferrites, the measured iron percentage was 64.8%, the resulting difference of 7% being Zn ions, assuming that the oxygen concentration is similar in both type of MNPs.

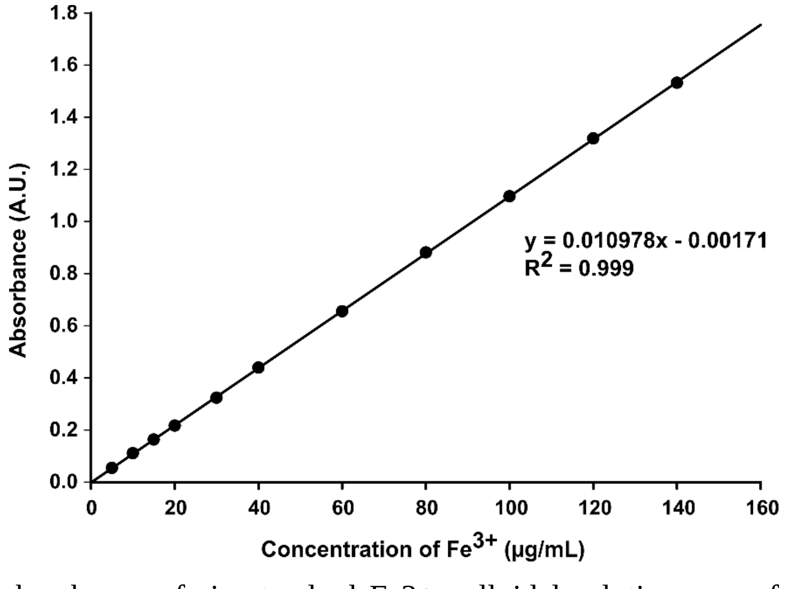

Figure S2. The absorbance of six standard Fe3+ colloidal solutions as a function on Fe3+ concentration measured at a λ = 490 nm. The values are expressed as mean ± SD of three replicates. The black line represents a linear regression of the experimental values.

S3. Magnetite (Fe_3O_4) nanoparticles:

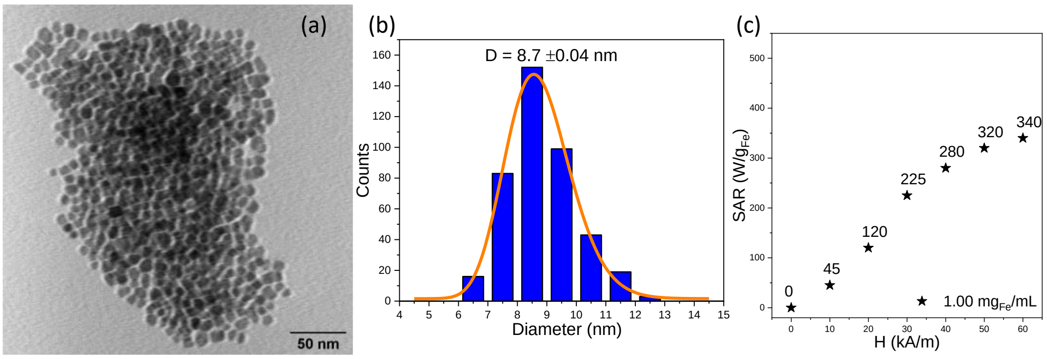

Figure S3. (a) TEM images of Fe_3O_4 nanoparticles. (b) Size distribution histogram of Fe_3O_4 nanoparticles fitted to a log-normal distribution (orange line). (c) Field dependence of Specific Absorption Rate (SAR) for Fe_3O_4 nanoparticles dispersed in water at 1.00 mg_{Fe}/mL.

S4. Thermosensitive Magnetoliposomes with different types of MNPs:

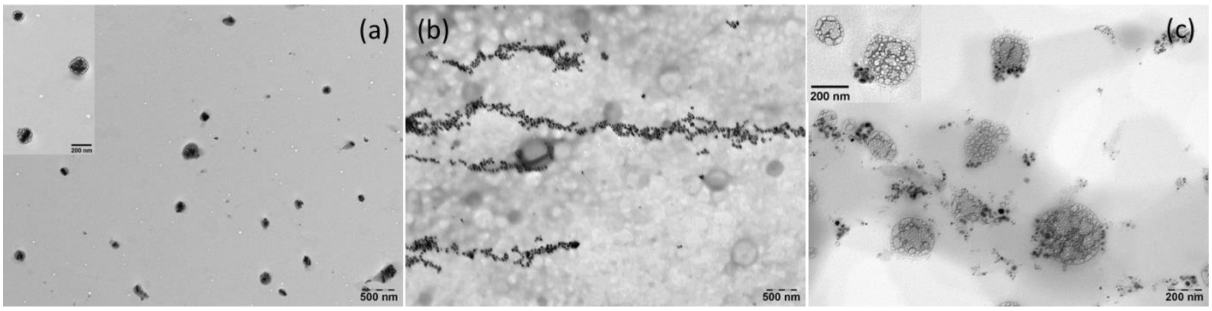

Figure S4. TEM images of TsMLs prepared with (a) superparamagnetic Fe_3O_4 nanoparticles, (b) 27 nm ferromagnetic Zn ferrites and (c) Zn ferrites coated with SiO_2 layer.

S5. Determination of internalized amount of doxorubicin inside the TsMLs and the released amount of doxorubicin either passive or via magnetic hyperthermia from TsMLs:

Upon preparation of TsMLs loaded with DOX, it results a mean volume of 17 mL. The TsMLs are then magnetically separated, resulting 1.5 mL of TsMLs and 15.5 mL of supernatant containing non-encapsulated DOX. For the determination of DOX concentration, the following protocols have been established as a function of initial amount of DOX used in preparation method:

1. h-DOX-TsMLs: 150 µL of supernatant was added to 1350 µL of ethanol and centrifugated for 5 min at 1500 rot/min. 1 mL of solution was then added to a UV-Vis cuvette and the absorbance at 450 nm was read following by automatic determination of DOX concentration based on the below calibration curve. The detected concentration was multiplied by 10 giving the DOX concentration in supernatant. This was further multiplied with 15.5 mL resulting the amount of non-encapsulated DOX. By subtraction from initial DOX amount (concentration multiplied by 17 mL), the internalized DOX amount can be found.

2. l-DOX-TsMLs: 75 µL of supernatant and 75 µL of stock DOX aqueous solution (300 µg/mL) was added to 1350 µL of ethanol and centrifugated for 5 min at 1500 rot/min. 1 mL of solution was then added to a UV-Vis cuvette and the absorbance at 450 nm was read following by automatic determination of DOX concentration based on the below calibration curve. The detected concentration was multiplied by 20 and by subtraction of 300 µg/mL, it resulted the DOX concentration in supernatant. This was further multiplied with 15.5 mL resulting the amount of non-encapsulated DOX. By subtraction from initial DOX amount (concentration multiplied by 17 mL), the internalized DOX amount can be found.

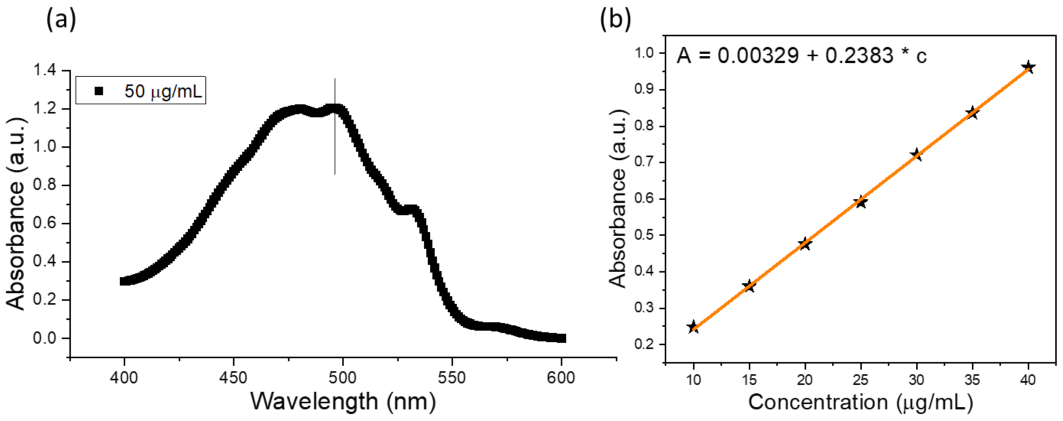

Figure S5. (a) UV-Vis spectrum of doxorubicin at a concentration of 50 µg/mL. The vertical line indicates the maximum of absorbtion band (495 nm) used in the determination. (b) Calibration curve of DOX – absorbance at 495 nm versus DOX concentration (10 to 40 µg/mL) – realized in ethanol as dispersion medium, used in the determination of DOX concentration in different experiments. The orange line represets the linear fit of experimental data.

S6. Magnetic hyperthermia properties of thermosensitive magnetoliposomes

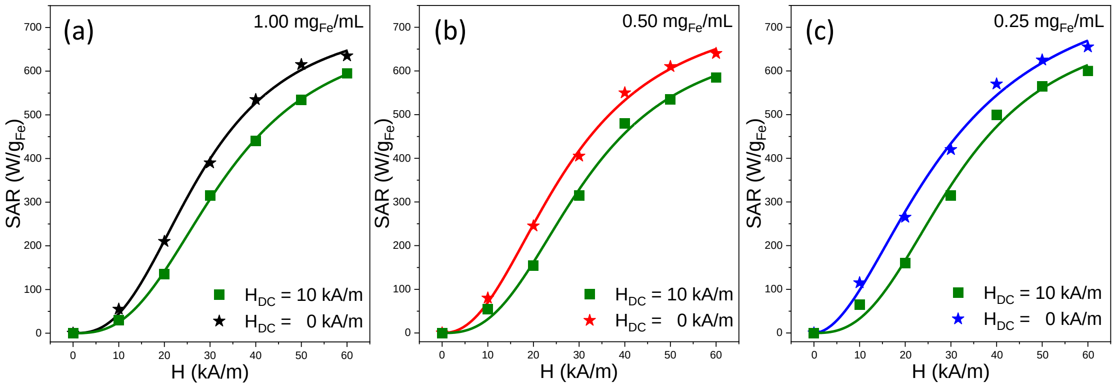

Figure S6. Field dependence of Specific Absorption Rate (SAR) for Zn ferrites confined in liposomes at concentration of (a) 1.00 mg$_{Fe}$/mL, (b) 0.50 mg$_{Fe}$/mL and (c) 0.25 mg$_{Fe}$/mL with and without a H_{DC} of 10 kA/m applied parallel with AFM lines.

Table S2. Fitting parameters of SAR evolution with H for Zn ferrites confined in liposomes with and without a H_{DC} applied parallel to AMF lines.

**Conditions**	**c (mg$_{Fe}$/mL)**	**SAR$_{max}$ (W/g$_{Fe}$)**	**H$_{cHyp}$ (kA/m)**	**Power Coefficient n**
H_{DC} = 0 kA/m	1.00	730	20.81	2.6
	0.50	760	18.09	2.3
	0.25	835	15.59	1.9
H_{DC} = 10 kA/m	1.00	705	25.08	2.8
	0.50	690	23.22	2.7
	0.25	725	23.41	2.6

S7. Cellular internalization of Zn ferrites and TsMLs:

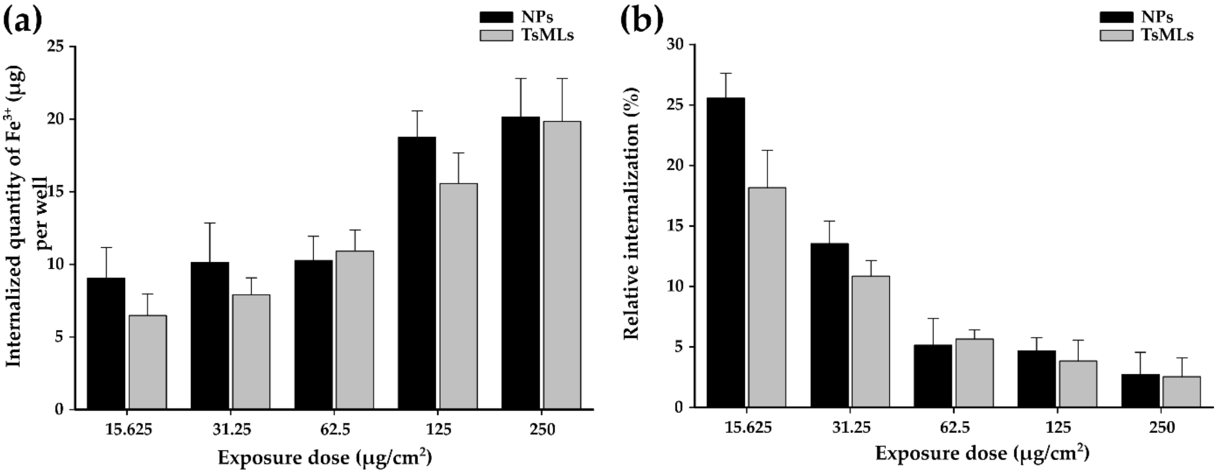

Figure S7. Cellular internalization of Zn ferrites and TsMLs in A549 after a 48 h exposure: (a) total iron amount per well and (b) the relative internalization. Values are expressed as mean ± SD of three biological replicates.
